# Senile Scleral Plaque Mimicking an Intraocular Foreign Body

**DOI:** 10.7759/cureus.78410

**Published:** 2025-02-03

**Authors:** Hamizah Muhammad, Wei Sheng Chan, Juanarita Jaafar, Wan-Hazabbah Wan Hitam

**Affiliations:** 1 Department of Ophthalmology and Visual Science, School of Medical Sciences, Universiti Sains Malaysia, Kubang Kerian, MYS; 2 Department of Ophthalmology and Visual Science, Hospital Universiti Sains Malaysia, Kubang Kerian, MYS; 3 Department of Ophthalmology, Hospital Sultanah Bahiyah, Alor Setar, MYS

**Keywords:** calcified plaque, intraorbital foreign body, ophthalmology trauma, sclera, trauma imaging

## Abstract

Senile scleral plaques are localized, calcified deposits typically occurring bilaterally in elderly patients and are often asymptomatic. They usually appear near the insertion of the rectus muscles and are associated with age-related changes in the sclera. In rare cases, a senile scleral plaque can occur unilaterally and may mimic the appearance of an intraocular foreign body (IOFB) especially in a post-traumatic case. We report a case of a unilateral senile scleral plaque.

A 65-year-old gentleman presented with left eye pain and blurring of vision while hammering a nail into the wall at home. On examination, his visual acuity was 6/24 in the right eye and 6/60 in the left eye. On his left eye, there was a conjunctival laceration at eight o'clock. His left anterior chamber was shallow with the presence of cells 3+. His left pupil was irregular with an area of sphincter tear at eight o'clock. His left lens was posteriorly dislocated. Computed tomography (CT) of the orbit revealed a hyperdense opacity temporally, which was suspicious of an intraocular foreign body. He underwent examination under anesthesia with phacofragmentation and pars plana vitrectomy for his posteriorly dislocated crystalline lens. Intraoperatively, there was the presence of a senile scleral plaque at the temporal region, with no evidence of an intraocular foreign body. He underwent a scleral fixated intraocular lens implantation later on, and postoperatively, his visual acuity for his left eye was 6/6. He remains well with good vision throughout his follow-up.

Although less common, the unilateral presentation of senile scleral plaques should be considered in the differential diagnosis of hyperdense orbital lesions in elderly patients. The potential for senile scleral plaques to mimic intraocular foreign bodies underscores the importance of thorough clinical evaluation and the careful interpretation of imaging studies in patients presenting with ocular trauma. A thorough clinical evaluation, coupled with a cautious interpretation of radiologic findings, is essential in guiding appropriate management and ensuring optimal visual outcomes.

## Introduction

Penetrating ocular injuries with an intraocular foreign body (IOFB) are one of the causes of visual impairment and require immediate management to prevent further damage and complications such as infection, permanent vision loss, or globe rupture. IOFB can present with a wide variety of clinical signs and symptoms, depending on the size, location, and material of the foreign body [1]. However, other pathologies such as a senile scleral plaque may resemble IOFB in imaging studies, which can lead to diagnostic challenges.

A senile scleral plaque is a localized area of scleral degeneration that usually occurs in the elderly due to actinic damage and tractional forces from extraocular muscle [2,3]. It usually appears as a grayish, depressed lesion on the sclera, often bilateral, and remains asymptomatic [4]. It is commonly located near the insertion of the rectus muscle. However, in rare cases, it can occur unilaterally and may mimic the appearance of an intraocular foreign body on computed tomography (CT) due to its hyperdense appearance on imaging. This similarity is especially challenging in the case of ocular trauma as the clinical suspicion of IOFB is high. This case report discusses the diagnostic challenges and management of a senile scleral plaque that presents similarly to an IOFB.

## Case presentation

A 65-year-old gentleman presented with left eye pain and blurring of vision while hammering a nail into the wall at home. It was associated with left eye redness and tearing. Otherwise, he denied any floaters or flashes of light. There was no history of previous ocular trauma or surgery.

On examination, his visual acuity was 6/24 in the right eye and 6/60 in the left eye. On his left eye, there was a conjunctival laceration at eight o'clock. His left anterior chamber was shallow with the presence of cells 3+. His left pupil was irregular with an area of sphincter tear at eight o'clock. His left lens was posteriorly located. His right eye anterior chamber examination was unremarkable apart from a nuclear sclerotic cataract. His fundus examination of both eyes otherwise was normal.

**Figure 1:**
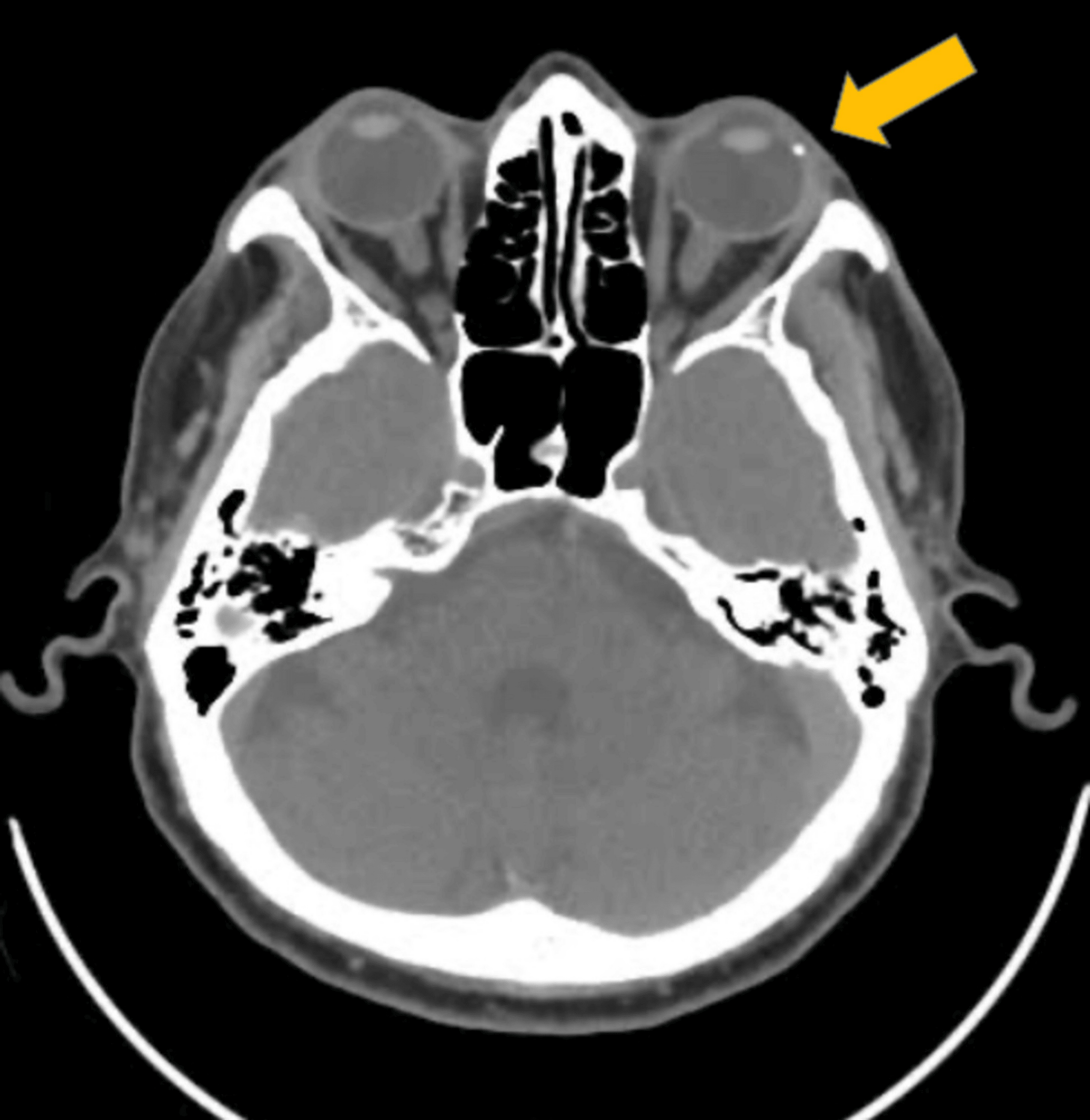
Computed tomography (CT) image showing a hyperdense lesion at the temporal globe of the left eye.

Computed tomography of the orbit revealed a hyperdense opacity temporally, which was suspicious of an intraocular foreign body (Figure 1).

**Figure 2:**
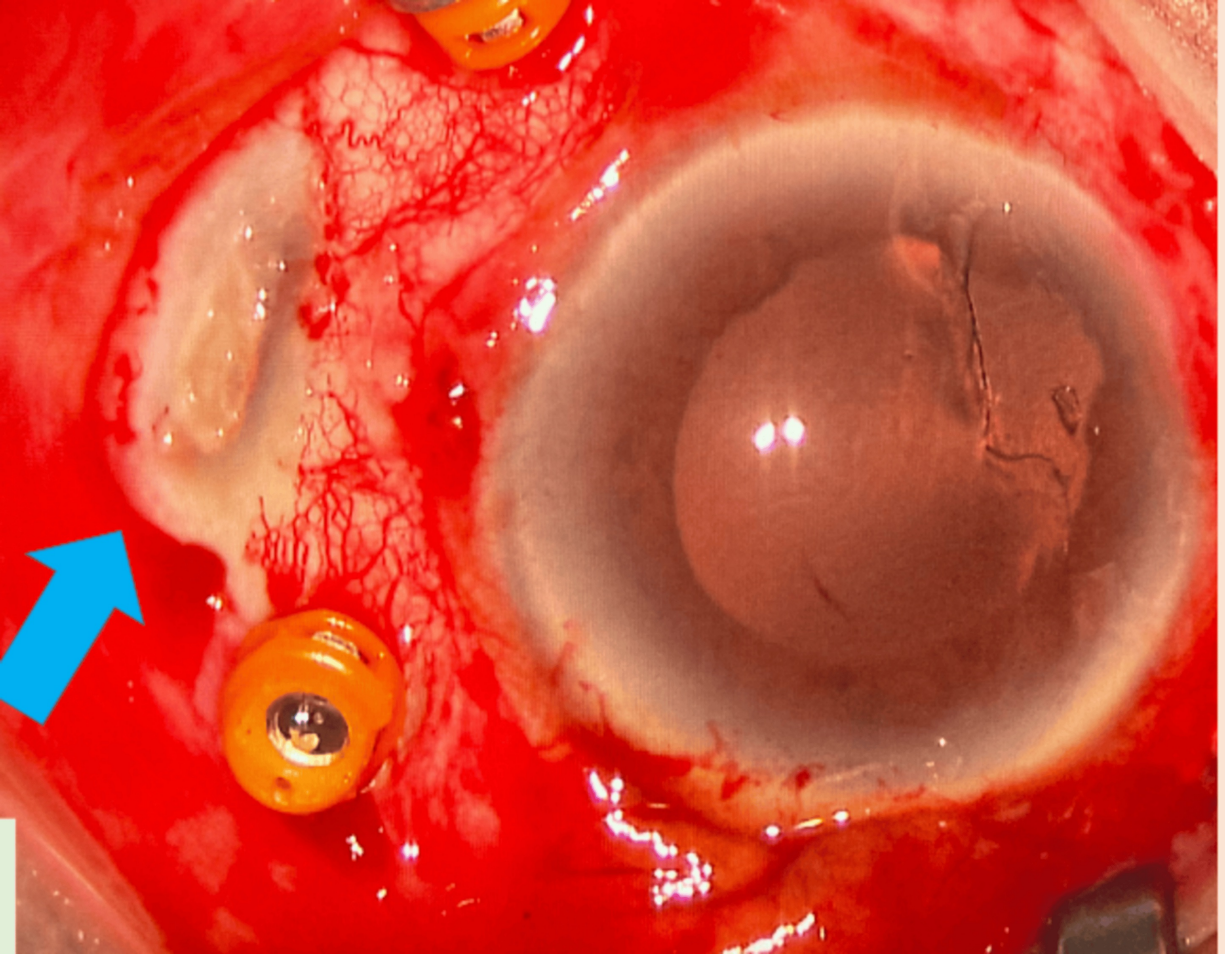
Grayish, depressed lesion on the sclera near the rectus muscle insertion, indicative of a senile scleral plaque.

He underwent examination under anesthesia with phacofragmentation and pars plana vitrectomy for his posteriorly dislocated crystalline lens. Intraoperatively, there was the presence of a senile scleral plaque at the temporal region, with no evidence of an intraocular foreign body (Figure 2). He underwent a scleral fixated intraocular lens implantation later on, and postoperatively, his visual acuity for his left eye was 6/6. He remains well with good vision throughout his follow-up.

## Discussion

Ocular trauma to the eye of a high-velocity nature may result in a penetrating ocular injury and a retained intraocular foreign body, if the said object remained lodged in the eye. Common symptoms of a retained intraocular foreign body would include eye pain, redness, and blurring of vision. Imaging studies, particularly computed tomography, play a crucial role in diagnosis, but the presence of a previously undiagnosed senile scleral plaque may be a red herring.

Senile scleral plaques are typically asymptomatic, bilateral, and located at the insertion of the rectus muscles [4]. However, they can occasionally present unilaterally and mimic the radiologic appearance of an IOFB due to their hyperdense, calcified nature on CT imaging. The appearance of these plaques as ovoid hyperdensities can lead to a diagnostic challenge, particularly in elderly patients with a history of trauma, where the clinical suspicion for an IOFB is high.

Commonly, senile scleral plaques are seen among the elderly, and their prevalence increases with age. It is reported that senile scleral plaques were observed in up to 20% of patients aged 80 years and above [4]. This higher prevalence may be attributed to the accumulation of degenerative changes in the sclera over time, which can occur without overt clinical symptoms [2].

The pathogenesis of senile scleral plaques is primarily attributed to age-related degenerative changes in the sclera. As individuals age, the sclera undergoes structural changes, including collagen degeneration and calcification, which can manifest as senile scleral plaques [3]. Furthermore, the mechanical stress over the scleral wall at the insertion point of the rectus muscle contributes to the localization of these lesions [5].

Diagnosing senile scleral plaques requires a combination of clinical examination and imaging studies. Optical coherence tomography (OCT) and computed tomography can be utilized to visualize scleral plaques and assess their characteristics. OCT can reveal the hyporeflective structure of the plaques, while CT may show calcifications associated with senile scleral plaques [5].

In this case, due to the nature and character of the trauma, the findings of a hyperdense lesion within the scleral wall raised the alarm on the possibility of an intraocular foreign body, which warranted surgical exploration. However, the intraoperative discovery of a senile scleral plaque instead of an IOFB underscores the importance of considering differential diagnoses when interpreting radiologic findings.

Distinguishing between an IOFB and a senile scleral plaque is essential for determining appropriate management. Although CT imaging is highly sensitive for detecting metallic intraocular foreign bodies, the presence of calcified structures such as scleral plaques can complicate the diagnostic process [6]. In this case, the patient's advanced age and the unilateral presentation of the scleral plaque added to the diagnostic challenge.

The management of a senile scleral plaque vastly differs from that of an intraocular foreign body. The management of penetrating ocular trauma with suspected IOFBs typically involves prompt surgical exploration, especially when imaging findings are suggestive [7]. However, when imaging raises the possibility of a non-foreign body etiology, such as a senile scleral plaque, careful consideration and correlation with clinical findings are essential to guide appropriate management. Further consultation with the radiologist should also be considered to assist in diagnosing potentially confounding cases.

## Conclusions

While senile scleral plaques are generally considered a benign condition, their ability to mimic more serious pathology due to their hyperdense nature on imaging, such as an intraocular foreign body, poses diagnostic challenges and hence requires careful clinical and radiologic evaluation. This case highlights the complexity of evaluating ocular trauma in elderly patients with preexisting senile scleral plaque, where such plaque can present as a hyperdense lesion on imaging, leading to diagnostic challenges.

Although less common, the unilateral presentation of senile scleral plaques should be considered in the differential diagnosis of hyperdense orbital lesions in elderly patients. The potential for senile scleral plaques to mimic intraocular foreign bodies underscores the importance of thorough clinical evaluation and careful interpretation of imaging studies in patients presenting with ocular trauma.
